# Effect of liver transplantation in combined hepatocellular and cholangiocellular carcinoma: a case series

**DOI:** 10.1186/s12885-015-1252-7

**Published:** 2015-04-08

**Authors:** Di Wu, Zhong-Yang Shen, Ya-Min Zhang, Jian Wang, Hong Zheng, Yong-Lin Deng, Cheng Pan

**Affiliations:** Department of Hepatobiliary Surgery, Orient Organ Transplant Center, The First Center Hospital of Tianjin, Tianjin, 300192 China

**Keywords:** Combined hepatocellular and cholangiocellular carcinoma, Liver neoplasm, Cholangiocarcinoma, Liver transplantation, Prognosis, Recurrence, Neoplasm recurrence, Local

## Abstract

**Background:**

Liver transplantation is a treatment option for combined hepatocellular and cholangiocellular carcinoma (cHCC-CC) but its prognostic significance remains unclear. The present study aimed to evaluate the therapeutic effects of liver transplantation on cHCC-CC and analyze the clinicopathological factors affecting prognosis.

**Methods:**

Retrospective analysis of the clinicopathological data of a case series of 21 patients with cHCC-CC who underwent orthotopic liver transplantation from April 2000 to April 2011 was performed. Cumulative survival rate and tumor-free survival rate were calculated using the Kaplan-Meier method followed by the log-rank test.

**Results:**

The operative survival rate of the 21 patients was 100%; the 30 day mortality was 4.8% (1/21) and 90-day mortality was 9.5% (2/21); 1-, 2-, 3-, and 5-year overall cumulative survival rates were 64%, 47%, 39%, and 39%, respectively; and the corresponding cumulative tumor-free survival rates were 64%, 37%, 30%, and 30%, respectively. Cumulative tumor diameter, lymph node metastasis, macroscopic portal vein tumor thrombus, and mixed states according to Allen typing were identified as the primary influencing factors of poor prognosis (all *P* < 0.05).

**Conclusion:**

Liver transplantation may be an effective therapeutic method for the treatment of cHCC-CC. Strict screening of potential liver transplantation candidates with cHCC-CC can help reduce the risks of tumor recurrence and metastasis.

## Background

Combined hepatocellular and cholangiocellular carcinoma (cHCC-CC) is an uncommon but discrete variant of primary liver cancer, with different biological behavior from hepatocellular carcinoma (HCC) and cholangiocellular carcinoma (CC). It accounts for 0.4–14% of all primary malignant liver tumors [1]. Histologically the tumor demonstrates features of both hepatocellular and cholangiocellular epithelial differentiation [2]. cHCC-CC is more common in males than in females [3]. The median survival of patients with non-surgical treatment is approximately four months; and the respective 1-, 3-, and 5-year survival rates have been shown to be 26.5%, 12.5%, and 9.2%, respectively [1]. The accompanying disease characteristics include hepatitis B virus/hepatitis C virus infection, liver cirrhosis, vascular thrombosis, and hilar lymph node metastasis. Some of the features of cHCC-CC such as association with hepatitis virus and portal vein thrombosis are similar to HCC [4], whereas other features such as poor blood supply to the tumor and early onset of hilar and retroperitoneal lymph node metastasis are similar to CC [5]. The clinical features of cHCC-CC may be related to the dominant component in the tumor body. When bile duct-derived cancer cells are absolutely dominant in the tumor body, cHCC-CC clinically manifests to be more similar to CC.

Therapeutic methods for cHCC-CC include conservative treatment, liver resection (hepatectomy), and liver transplantation [3]. Other methods including local ablation (e.g., ethanol injection, microwave coagulation, and radiofrequency ablation [RFA]) and transcatheter arterial chemoembolization (TACE) have proved effective only in a small number of cases [6]. Local treatment methods such as TACE and percutaneous ethanol injection may have poor efficacy because of abundant interfibrillar substances and the poor blood supply found in cHCC-CC [7]. Liver resection can prolong the survival of a patient with cHCC-CC who has an early tumor stage and liver function that is tolerant to resection, and has achieved postoperative median survival times of 20 to 47 months [1].

Liver transplantation provides an option for the treatment of cHCC-CC in patients intolerant to liver resection [8]. The advantages of liver transplantation are better dissection of lymph nodes, elimination of background diseases of liver cancer, and no postoperative risk of liver dysfunction due to liver resection. Patients with cHCC-CC receiving liver transplantation have shown better survival than the same-stage patients with cHCC-CC undergoing hepatectomy [3,8]. Similarly, liver transplant recipients with HCC have better survival rates than patients with cHCC-CC [9].

The clinical outcome of liver transplantation in patients with hepatocellular carcinoma (HCC) is well defined. But, there are fewer clinical reports available on liver transplantation in the treatment of cHCC-CC, and factors relevant to its prognosis remain unclear. The present study aimed to explore the clinical efficacy of liver transplantation in patients with cHCC-CC and evaluate the effects of different clinicopathological factors on prognosis of cHCC-CC. This information should add important data to that already available on the best treatment option for patients with cHCC-CC and whether liver transplant should be selected.

## Methods

### Clinical data

From April 2000 to April 2011, a cases series of 21 patients with cHCC-CC who underwent orthotopic liver transplantation (OLT) were selected from the First Center Hospital of Tianjin (Tianjin, China). This study was approved by the ethics committee of the First Center Hospital of Tianjin (E2014008L) and complied with the Declaration of Helsinki, and all participants provided written informed consent.

The cases included male patients whose diagnoses were pathologically confirmed as cHCC-CC with an age of onset of 35 to 65 years (mean age, 53 years). Twenty patients had hepatitis (fifteen with hepatitis B, four with hepatitis C, and one with hepatitis B and C) and one patient did not have hepatitis. According to the Child-Pugh classification of severity of liver disease, patients with cHCC-CC were classified as follows: sixteen patients with Grade A liver disease, three patients with Grade B liver disease, and two patients with Grade C liver disease. Prior to liver transplantation, 13 patients had not received preoperative adjuvant therapy. While eight patients had received preoperative adjuvant therapy including TACE and/or radiofrequency ablation (RFA, n = 7) and chemotherapy pump placement (n = 1). The patients’ preoperative serum α-fetoprotein (AFP) level (n = 13) was >20 ng/mL and preoperative carbohydrate antigen 19–9 (CA 19–9) level (n = 12) was ≥ 37U/mL.

Intraoperatively resected liver tissues were used for routine histopathological examinations. In the 21 cases 18 patients had cirrhosis while the remaining three patients had no cirrhosis. Five patients had single tumors, eight patients had 2 to 3 tumors, and eight patients had ≥ 4 tumors. Twelve patients had tumor lesions in the right lobe, while the remaining nine patients had tumor lesions in both the right and left lobes of the liver. Cumulative tumor diameter (the maximal diameter of single tumors or the sum of tumor diameters for multi-tumors) was ≤5 cm in four patients, 5 to 10 cm in seven patients, and >10 cm in ten patients. Patients also had accompanying diseases such as hilar lymph node metastasis (n = 5), macroscopic portal vein tumor thrombus (n = 8), and microvascular thrombosis (n = 14). The component in HCC was moderately or well differentiated in sixteen patients and poorly differentiated in five patients. The component in CC was moderately or well differentiated in twelve patients and poorly differentiated in nine patients. According to the typing method of Allen et al., [10] there were twelve patients in the separation state, three patients in the collision state, and six patients in the mixed state.

All 21 patients underwent conventional non-bypass OLT from a cadaver donor of the same blood type. Postoperatively, the patients received a standard triple immunosuppressive therapy with hormone + tacrolimus (FK506) + mycophenolate mofetil (CellCept®) for three months. Thereafter, tacrolimus (FK506) was administered individually to maintain blood trough concentration at 3 to 6 ng/mL.

### Follow-up

All patients were either hospitalized or visited as outpatients for follow-up. There were no patients lost to follow up. The patients were re-examined postoperatively by abdominal ultrasound, chest x-ray, and blood tests (serum AFP and CA 19–9 measurements) monthly, and at six months and quarterly thereafter. The patients underwent a chest and abdominal computed tomography scan, and bone scan for diagnosis of tumor recurrence. The time and cause of death were followed up.

### Statistical analysis

Data were statistically analyzed using SPSS, version 15.0 (SPSS Inc., Chicago, IL, USA). Cumulative survival rate and tumor-free survival rate were calculated using the Kaplan-Meier method, followed by the log-rank test for univariate analysis. A *P-*value of less than 0.05 was considered statistically significant.

## Results

### Follow-up results

As of April 2013, 14 recipients of liver transplants survived over a year. In the 21 case series, the operative survival rate of the patients was 100%, the 30 day mortality was 4.8% (1/21) and 90-day mortality was 9.5% (2/21). One patient died due to an aneurysm rupture and bleeding during the first postoperative month. Another two patients died due to lung infection two and four months postoperatively. 8 of the 21 patients recurred or died of procedure related events within 6 months of transplant. Among these 8 patients, 3 had lymph nodes metastases.

**Figure 1:**
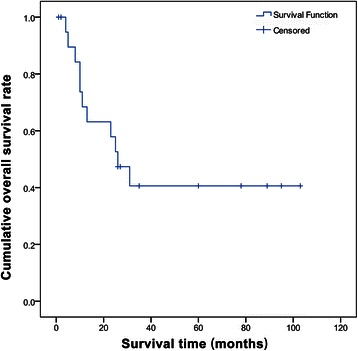
**Cumulative 1-, 2-, 3-, and 5-year overall survival of patients with combined hepatocellular and cholangiocellular carcinoma after liver transplantation.** Overall survival: 1-year overall survival 64%, 2-year overall survival 47%, 3-year overall survival 39%, 5-year overall survival 39%.

The cumulative 1-, 2-, 3-, and 5-year overall survival rates of the 21 patients with cHCC-CC were 64%, 47%, 39%, and 39%, respectively (Figure 1).

**Figure 2:**
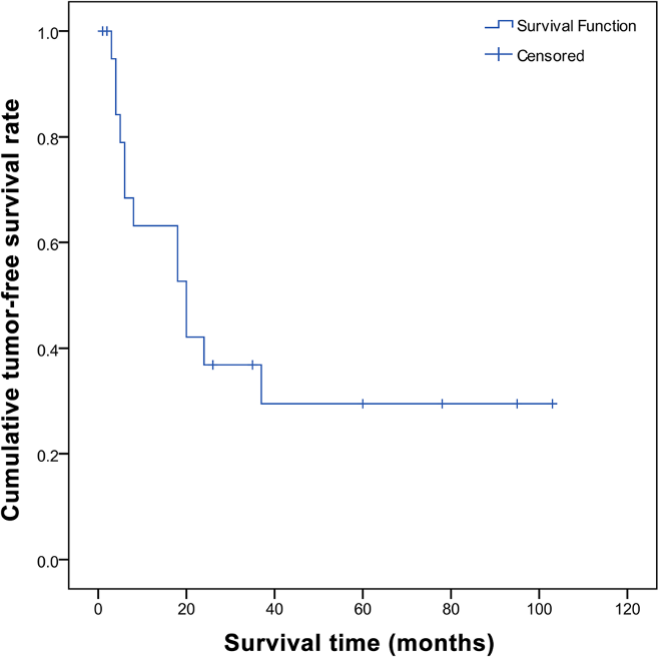
**Cumulative 1-, 2-, 3-, and 5-year tumor-free survival of patients with hepatocellular and cholangiocellular carcinoma after liver transplantation.** Tumor-free survival: 1-year tumor-free survival: 64%, 2-year tumor-free survival: 37%, 3-year tumor-free survival: 30%, 5-year tumor-free survival: 30%.

The cumulative 1-, 2-, 3, and 5-year tumor-free survival rates of the patients with cHCC-CC were 64%, 37%, 30%, and 30%, respectively (Figure 2).

The median survival time of the 21 patients with cHCC-CC was 23 months.

### Univariate analysis

The univariate analysis of relevant clinicopathological factors showed that the influencing factors of poor prognosis included cumulative tumor diameter, lymph node metastasis, macroscopic portal vein tumor thrombus, and mixed states according to Allen type (all *P* < 0.05) (Table 1).

**Table 1:** **Effects of different clinicopathological factors on the survival of patients with combined hepatocellular and cholangiocellular carcinoma after liver transplantation**

Prognostic factors	Cases (%)	Mean survival time (month)	*P-*value
Age			
<50-year	7 (33%)	50.8 ± 18.1	0.899
≥50-year	14 (67%)	45.0 ± 11.8	
Preoperative treatment			
None	13 (62%)	46.7 ± 12.3	0.769
TACE, RFA, or chemotherapy	8 (38%)	49.0 ± 14.1	
Preoperative AFP			
<20 ng/mL	8 (38%)	44.0 ± 17.2	0.898
≥20 ng/mL	13 (62%)	37.1 ± 9.5	
Preoperative CA19-9			
<37U/mL	9 (43%)	53.6 ± 15.8	0.268
≥37U/mL	12 (57%)	48.8 ± 8.9	
Child-Pugh Grading			
Grade A	16 (76%)	47.3 ± 13.1	0.347
Grade B	3 (14%)	39.1 ± 9.1	
Grade C	2 (10%)	53.6 ± 12.0	
Cirrhosis*			
No	3 (14%)	9.0 ± 1.0	0.031
Yes	18 (86%)	52.8 ± 11.1	
Tumor distribution			
Right lobe	12 (57%)	53.5 ± 12.9	0.265
Double lobes	9 (43%)	41.3 ± 10.2	
Cumulative tumor diameter*			
≤5 cm	4 (19%)	83.5 ± 16.9	0.047
5–10 cm	7 (33%)	32.8 ± 10.1	
>10 cm	10 (48%)	17.7 ± 4.0	
Tumor quantity			
Singular	5 (24%)	37.3 ± 19.2	0.084
2–3	8 (38%)	50.3 ± 14.8	
≥4	8 (38%)	34.3 ± 13.0	
Lymph node metastasis*			
Yes	5 (24%)	14.0 ± 6.1	0.039
No	16 (76%)	54.7 ± 11.7	
HCC differentiation			
Intermediate to well differentiated	16 (76%)	54.3 ± 15.2	0.074
Poorly differentiated	5 (24%)	45.6 ± 12.1	
CC differentiation			
Intermediate to well differentiated	12 (57%)	56.7 ± 9.3	0.083
Poorly differentiated	9 (43%)	41.0 ± 13.8	
Macroscopic thrombosis*			
Yes	8 (38%)	21.0 ± 5.1	0.028
No	13 (62%)	57.9 ± 13.1	
Microvascular thrombosis			
Yes	14 (67%)	42.5 ± 12.5	0.226
No	7 (33%)	58.8 ± 14.9	
Allen typing*			
Separation/collision	15 (71%)	47.9 ± 9.9	0.037
Mixed	6 (29%)	21.3 ± 14.2	

*Note*: AFP = alpha-fetoprotein; CA19-9 = carbohydrate antigen 19–9; CC = cholangiocellular carcinoma; HCC = hepatocellular carcinoma; RFA = radio frequency ablation; TACE = transcatheter arterial chemoembolization.

* by the log-rank test, *P <* 0.05.

### Baseline characteristics of patients whose tumor recurred within 1 or 2 years

Of the 21 patients, 7 showed recurrence of tumors during the first year (33.3%) and 11 within 2 years (52.4%) (Table 2). The patients that had tumor recurrence within 1 year showed mean tumor sizes of 11.86 ± 2.90 cm in diameter and within 2 years 11.00 ± 4.13 cm in diameter. Patients whose tumor recurred within 1 or 2 years had similar presence of lymph node metastases, 2/7 (28.6%) and 3/11 (27.3%), respectively. Of the patients with tumor recurrence within 2 years, 9 (81.8%) had cirrhosis (Table 2).

**Table 2:** **Baseline characteristics of patients with combined hepatocellular and cholangiocellular carcinoma after liver transplantation that had tumor recurrence within 1 or 2 years**

Patients (n)	Age (years)	Free- tumor survival time (months)	Survival time (months)	Cumulative tumor diameter (cm)	Tumor quantity	Lymph node metastasis	Macroscopic thrombosis	Microvascular thrombosis	Cirrhosis
Recurred within 1 year									
2	61	5	10	14	3	No	No	Yes	Yes
4	54	4	10	8	1	No	No	Yes	No
5	59	8	13	12	5	Yes	No	Yes	Yes
7	43	6	11	7	1	No	No	No	Yes
8	35	3	5	15	6	No	Yes	Yes	Yes
10	40	4	4	13	5	Yes	Yes	Yes	Yes
20	55	6	8	14	2	No	No	Yes	No
Recurred within 2 year									
9	53	18	25	4	1	Yes	No	Yes	Yes
13	49	20	27	10	6	No	No	Yes	Yes
14	59	18	23	6	2	No	Yes	Yes	Yes
17	58	20	26	18	2	No	Yes	No	Yes

## Discussion

Although relatively rare, cHCC-CC accounts for 0.4–14% of primary liver cancers [1]. There is some evidence that liver transplantation may be the best available treatment for improving survival rates [3,9]. The aim of this investigation was to retrospectively assess the outcomes of patients with cHCC-CC undergoing liver transplant and to investigate the factors that might be involved in the prognosis of the patients. We found that the five-year overall survival rate was 39%. Univariate analysis suggests that prognosis is related to the clinicopathological factors of cumulative tumor diameter, lymph node metastasis, macroscopic portal vein tumor thrombus and mixed states according to Allen type.

In the present study, the cHCC-CC disease manifestations of the 21 male patients were as follows: combined hepatitis accounted for 95% (20/21), cirrhosis accounted for 86% (18/21), vascular invasion accounted for 67% (14/21), and lymph node metastasis accounted for 24% (5/21). The present study results are consistent with the data previously reported in the literature [6,11,12]. Kassahun and Hauss [1] comprehensively analyzed 424 cases of cHCC-CC in eighteen reports and indicated that hepatitis virus infection plays an important role in the pathogenesis of cHCC-CC. However, any differences in the mechanisms underlying the pathogenesis of HCC and cHCC-CC remain unclear.

In the present study, postoperative cumulative 1-, 2-, 3-, and 5-year survival rates of patients with cHCC-CC treated by liver transplantation were 64%, 47%, 39%, and 39%, respectively; and the corresponding cumulative tumor-free survival rates were 64%, 37%, 30%, and 30%. The overall survival rate of patients who underwent surgical OLT was better than the rates for non-surgical treatment as reported previously in the literature [6]. And the five-year overall survival was similar to previous studies that have evaluated this measure although lower than recent reports of 41% [3], 50% [13] and 60% [14] it was better than another at 16% [15]. These values are generally from small populations of patients because of the rarity of this disease so the values may reflect different patient pathological factors.

Tumor size and number, vascular invasion, and lymph node metastasis are important prognostic factors of liver transplantation in HCC [16,17]. In the present study the results of the univariate analysis showed that the cumulative tumor diameter, macrovascular invasion, and lymph node metastasis significantly affected the postoperative survival of patients with cHCC-CC (all *P* < 0.05). Preoperative diagnosis of lymph node metastasis is difficult due to a lack of specificity in imaging studies. This is because lymphadenopathy may also be caused by reactive hyperplasia of lymph nodes. Thus, it is necessary to perform preoperative or intraoperative liver biopsy in suspected cases. For instance, in the present study the mixed states according to Allen typing posed significantly different influences on the prognosis from other types. These data suggest that mixed-state tumors may be more invasive than the other two types of tumor. The present study results also found that 9 (81.8%) of the patients with recurrence within 2 years, had cirrhosis, this is perhaps associated with tumor development. Due to a lack of clinical cases, the above mechanism needs to be explored in future studies.

Three patients who died early post operatively had positive lymph nodes. One patient with lymph node metastasis died within 13 months and another patient with lymph nodes metastasis died within 25 months. 8 of the 21 patients had disease recurrence or died of procedure related events within 6 months of transplant. Among these 8 patients, 3 patients had lymph node metastases. This suggested that the effect of lymph node metastasis was very important. Panjala et al. reported that the 5-year survival rate after liver transplantation was only 16% in 12 patients with cHCC-CC without perineural or adjacent lymph node metastasis [15]. Because patients with lymph node metastasis had a poorer survival rate, we recommend freezing a section of suspicious lymph nodes at the time of surgical exploration or diagnostic laparoscopy prior to performing a transplant and the information this provides should alter the decision of whether to transplant or not. Careful selection of patients without lymph node metastasis may help to improve survival rates and reduce tumor recurrence rates in patients with cHCC-CC after liver transplantation.

A significant limitation of this study is the relatively few cases of cHCC-CC that were encountered and suitable for liver transplant, necessarily resulting in a small sample size. This may introduce some bias in the analysis of factors related to patient prognosis, giving some unexpected results such as longer term survival of the patients with cirrhosis of the liver.

## Conclusion

In conclusion, liver transplantation may be an effective clinical therapy with prolonged survival for a subgroup of patients with cHCC-CC. However, recurrence within the first two years is common, suggesting the need to develop selection criteria for OLT in this pathologic entity. The current study considers the effects of cumulative tumor diameter, vascular invasion, lymph node metastasis, and the type of cHCC-CC. Rigorous preoperative assessment of these criteria could enhance patient selection for OLT associated with cHCC-CC, thereby leading to reduced postoperative tumor recurrence and improved survival.

## Abbreviations

cHCC-CC: Combined hepatocellular and cholangiocellular carcinoma
OLT: Orthotopic liver transplantation
AFP: Alpha-fetoprotein
CA19-9: Carbohydrate antigen 19–9
CC: Cholangiocellular carcinoma
HCC: Hepatocellular carcinoma
RFA: Radio frequency ablation
TACE: Transcatheter arterial chemoembolization
